# Intraperitoneal pelvic leiomyoma with atypical location in an old man: The role for MRI in the differential diagnosis

**DOI:** 10.1016/j.radcr.2024.10.119

**Published:** 2024-11-22

**Authors:** Davide Turilli, Marco Anania, Vincenzo Marras, Claudia Pinna, Leandra Piscopo, Michele Obinu, Rosita Comune, Alberto Porcu, Mariano Scaglione, Salvatore Antonio Masala

**Affiliations:** a Radiology Department of Surgery, Medicine and Pharmacy, University of Sassari, Viale S. Pietro, Sassari, Italy; b Department of Medicine, Surgery and Pharmacy, University of Sassari, Sassari, Italy; c Department of Biomedical Sciences, Institute of Pathology, University of Sassari, Sassari, Italy; d UO Diagnostica per Immagini Ospedale San Francesco Nuoro, Nuoro, Italy; e Division of Radiology, Università degli Studi della Campania Luigi Vanvitelli, Naples, Italy

**Keywords:** Pelvic peritoneal masses, Leiomyoma, Magnetic resonance imaging

## Abstract

Primary pelvic peritoneal masses, not arising from major organs, are uncommon in adults. Leiomyomas are a group of benign smooth muscle tumors, most commonly found in the uterus in premenopausal women (70–80%). Extra-uterine locations are very rare and more frequent in women. We highlighted the role of MRI due to its capability in soft tissue characterization, that may positively impact the therapeutical decision-making process. Herein, we present the case of a 66-year-old man with a peritoneal solid mass suspicious for a leiomyoma at Magnetic Resonance Imaging and confirmed at histologic specimen in order to discuss the crucial imaging findings that could raise suspicion of such a rare pathology in man.

## Introduction

Primary neoplasms that originate in the soft tissue of the pelvic intra-, sub-, or retroperitoneum in adults, outside solid organs and hollow viscera, are very rare; they occur in both men and women and affect a wide age range, although they often prefer the latter during the reproductive age [[1], [2], [3], [4], [5]]. These tumors can arise from any of the anatomic components of these spaces, such as nerves, lymph nodes, connective tissue and vascular and lymphatic vessels [6]. Therefore, it results in a wide range of neoplasms [2,7]. These tumors are very rare, therefore their correct diagnostic classification is very difficult. It is influenced by clinical examination and/or a preoperative imaging and the lack of familiarity of physicians with the anatomy of this region, especially in men. They are often asymptomatic or manifest, often late, with nonspecific clinical features, mainly consequential to its mass effect, as abdominopelvic distention, pain or pelvic organ dysfunction [6,8]. Ultrasound (US) usually represents the first imaging approach, but is often inconclusive [1,7,9]. Magnetic Resonance Imaging (MRI) is the modality of choice for the diagnosis and evaluation of these masses [1,9]. Particularly, MRI offers a better spatial resolution compared to computed tomography (CT), resulting in a more detailed delineation of normal and pathologic spaces and structures, the extent of the neoplasm, and the staging of disease [5]. Furthermore, based on certain imaging features, multiparametric MRI can play a crucial role in the classification and characterization of these lesions. Particularly in the retroperitoneal space it allows a subdivision into cystic, fat-containing, calcified, vascular/hypervascular and mixoid lesions [2]. In intraperitoneal space characterization of tumors is often a more difficult challenge, since there is an overlap of radiological features between primary and secondary tumors and between benign and malignancy, but MRI is frequently fundamental in identifying the organ of origin of metastases (f.e. ovary) or in detecting non-neoplastic pathologies such as endometriosis [4,9]. Only a few cases are reported in the literature of peritoneal but especially retroperitoneal leiomyomas, which occur almost exclusively in premenopausal women [[10], [11], [12]]. In this article, we report the case of a very rare peritoneal pelvic leiomyoma in an adult old man, in which MRI allowed the correct diagnosis and consequent appropriate surgical planning.

## Case report

**Fig. 1:**
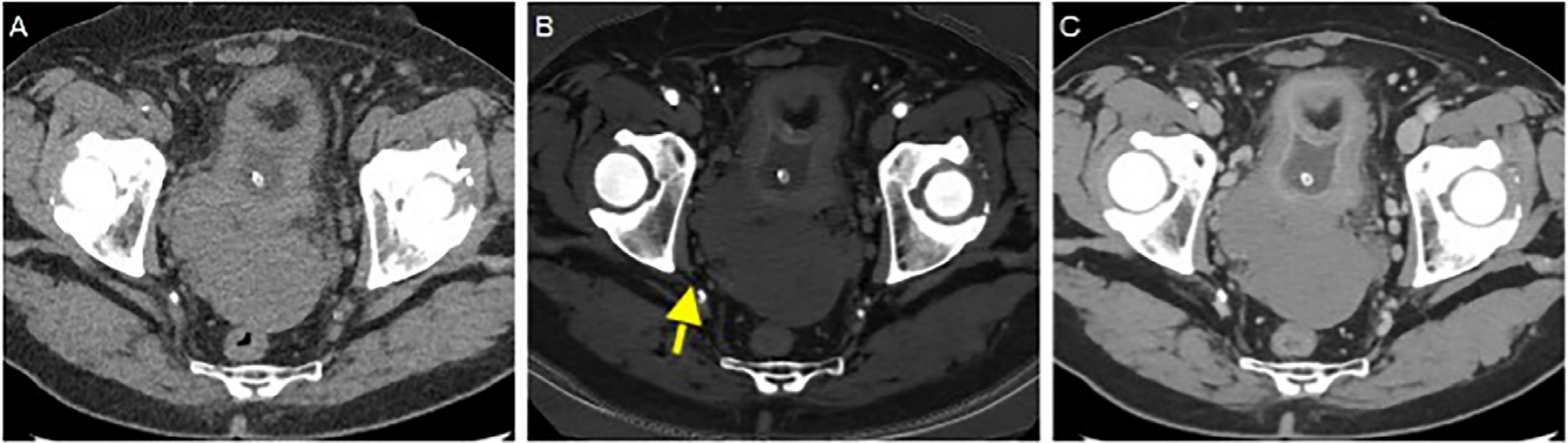
Axial non contrast (A), arterial Dual Energy Iodine Water (B, arrow) and portal phase contrast (C) pelvic computed tomography, show a voluminous, polylobulated, solid mass with smooth margin (yellow arrow), between bladder (with a catheter) and rectum in the median-paramedian bilateral area but prevalent on the right, well-limited, with regular margins, homogeneously hypodense and with mild contrast enhancement prevalent in portal phase.

**Fig. 2:**
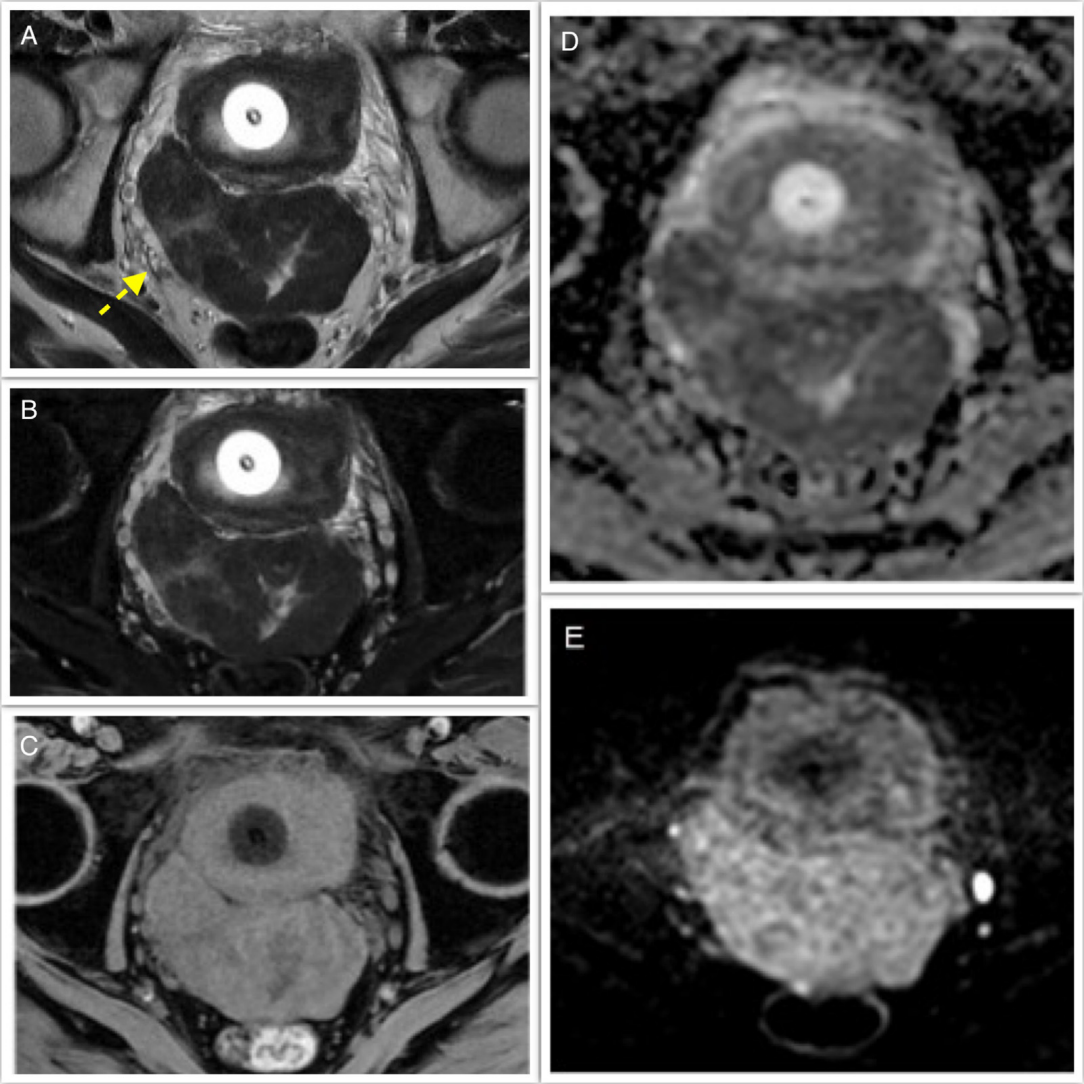
Axial Pelvic MRI T2-w with (A, arrow) and without (B) fat suppression, T1-w fat saturation (C) and DWI with ADC map (D-E), show a slight inhomogeneity of the lesion, that exhibit an hypointensity on T2, an hypointensity on T1 and a restriction of diffusion.

**Fig. 3:**
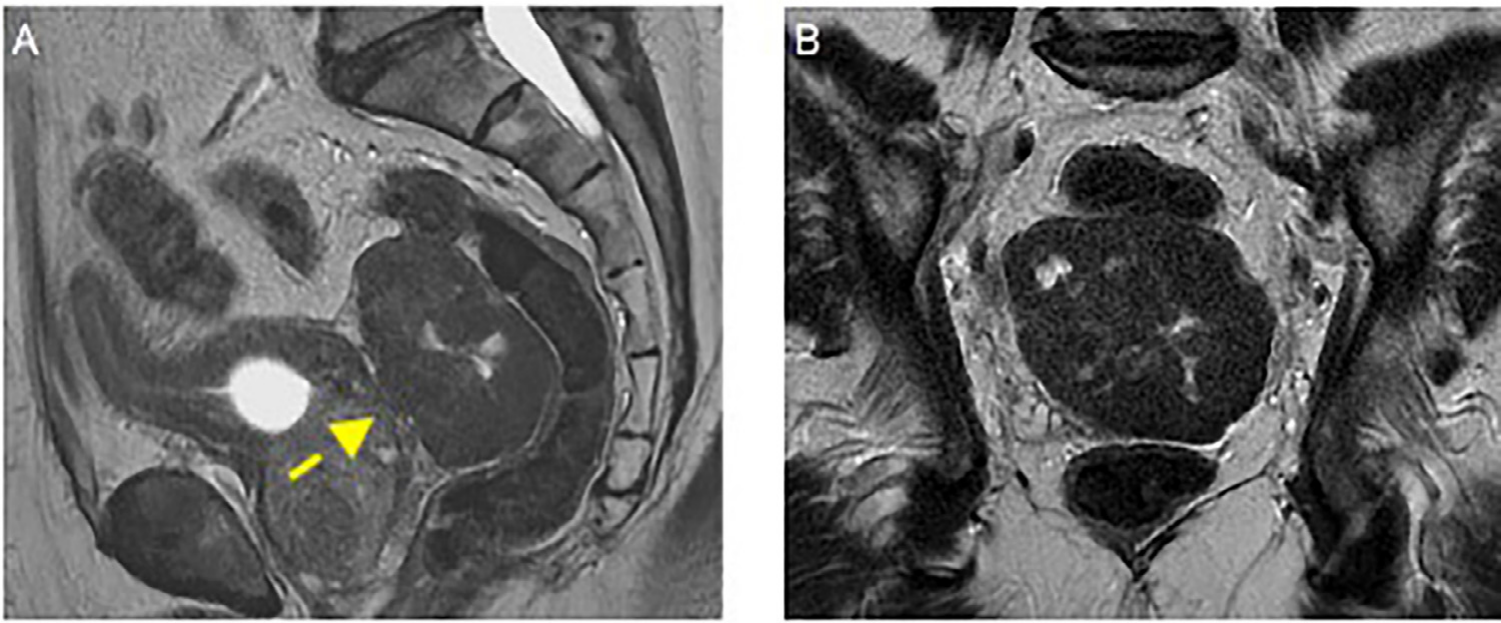
Sagittal (A, arrow) and coronal (B) Axial Pelvic MRI T2-w, reveal relationship with the main solid and hollow peritoneal organs, from which it does not originate.

**Fig. 4:**
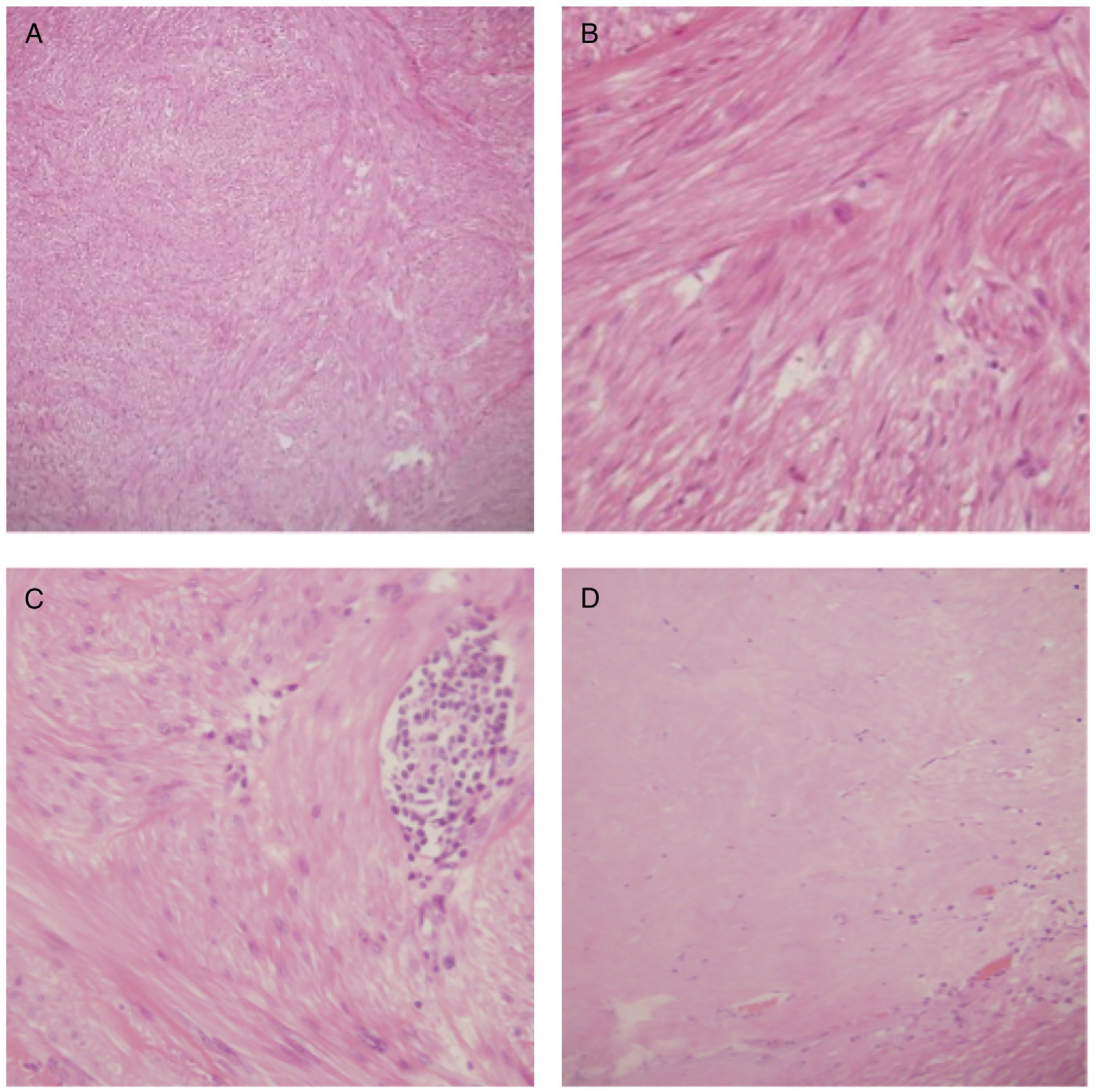
Ematossilina-eosina staining 10x (A): Bundles of spindle cells intertwined each other's (so called storiform pattern), 40x (B) The stromal cells appear as well differentiated cells with central nucleus and eosinophilic cytoplasm, 40x (C) Clusters of lymphocytes and mast cells are present among stromal cells, 20x (D) Sclerotic and hyaline regressive area totally acellular.

A 66-year-old man, with a history of type II diabetes mellitus, systemic hypertension and endoscopic polypectomy for a colic tubular adenoma with low-grade dysplasia. He complained dysuria and strangury, he had an indwelling bladder catheter for chronic urine retention. Clinical examination and laboratory panel were unremarkable. For these reasons, he previously performed abdominal ultrasound in another hospital that was nonconclusive. Because of persistent suspicious of an undetermined pelvic lesion, an abdominal-pelvic with contrast enhancement Computered Tomography (CT) was performed. A CT multiphase protocol was performed (noncontrast, arterial, corticomedullary, parenchymal, and excretory phases) with intravenous contrast (1.0–1.5 mL/kg injected at 3.5 mL/s, followed by 50 mL of a saline bolus injection). CT after contrast revealed a large irregular solid mass with smooth margin in the Douglas’ pouch (Fig. 1), with no signs of tumor invasion, the fat planes and the rectus-vescical cleavage were normal. The pelvic lymphadenopathy was regular. Subsequently, to better characterized the lesion, an MRI of the pelvis, were performed. The MRI showed hypointensity on T2-weighted (T2-w) images and the lack of an origin from the main peritoneal organs (Figs. 2, 3). The T1-weighted image revealed hypointensity and the diffusion-weighted image (DWI) showed a restriction. Then, the patient was hospitalized and was taken to the operating room, an “en block” resection was made, and the mass was well cleavable from the adjacent organs. No intra- or postprocedural complications occurred. Macroscopically, the lesion appeared as a circumscribed, solid nodular mass. The histological picture was characterized by a proliferation of stroll spindle cells organized in bundles intertwined with each other (Fig. 4A) with a clearly visible central nucleus and eosinophilic cytoplasm (Fig. 4B). Cytological atypia and mitosis were not present. Mixed with the stroll population were aggregates of lymphocytes and mast cells (Fig. 4C), and focally regressive areas were observed with the formation of acellular sclerotic hyaline bands (Fig. 4D). This histological exam is compatible with a fibro-leiomyoma.

## Discussion

The peritoneum is a thin, translucent mesothelial membrane, the largest and most complex of the serous membranes in the human body, formed by 2 layers, the parietal layer that lines the abdominal walls and the visceral layer that partially or completely covers the surface of solid organs and hollow viscera, creating a virtual space between them called the peritoneal cavity, which appears to be devoid of organs [3,12,13]. The other space identified by the peritoneum is represented by the subperitoneum, which is located deeper than itself. Therefore, all the abdomino-pelvic organs and their respective vessels, nerves and lymphatics are located in the subperitoneal space and in particular, those situated deeply in the posterior peritoneum are defined as retroperitoneal [14]. These spaces are separated from each other [14]. In the evaluation of an expansive abdominal lesion, the first challenge in the small ones is their detection, while in larger lesions, it is the correct anatomical space classification and/or recognition of the organ origin. Particularly primary pelvic masses in adults that originate outside solid organs and hollow viscera are rare but can arise from various cells or tissues, both retro- and intraperitoneally. The peritoneum is composed of mesothelial cells and a submesothelial layer of connective tissue composed of collagen, elastic fibers, fibroblast-like cells, vessels and lymphatics [15]. Therefore, neoplasms can arise from mesothelial cells, submesothelial mesenchymal cells, and uncommitted stem cells [15]. Peritoneal neoplasms can be classified into primary and secondary. The latter include neoplastic repetitive injuries such as carcinomatosis, pseudomixoma peritonei and lymphomatous, but also non neoplastic diseases as infectious and postinfectious lesions and a miscellany of pathologies such as endometriosis, splenosis, etc. Ptimary neoplasm includes mesothelial, epithelial, smooth muscle tutor and neoplasm of uncertain origin [4]. Multiparametric MRI can play a crucial role in the classification and characterization of these lesions. Leiomyoma is a benign mesenchymal tumor derived from smooth muscle. Their distribution is proportional to the distribution of smooth muscle tissue in the body [10]. Therefore, uterine leiomyomas are the most common gynecologic neoplasm (are found in 20% of women of reproductive age), followed by the gastrointestinal tract [[16], [17], [18]]. Furthermore, leiomyoma represents 3.8% of benign soft tissue tumors [19], and those located in deep soft tissue are uncommon and are classified into 2 subtypes: somatic soft tissue and retroperitoneal/abdominal ones. The former affect both genders equally and develop in the extremities, mainly in the thigh [11,12]. The latter affect almost exclusively premenopausal women, and they mostly originate in the pelvic retroperitoneum [11,12]. Disseminated peritoneal leiomyomatous [20,21] and parasitic leiomyoma [22] are the variants of extra-uterine leiomyoma that are mainly found within the peritoneal cavity, but isolated cases are also reported in the literature [18,[23], [24], [25]], mainly in women. So, their preoperative diagnosis is difficult, especially for males. Thus, after having identified the lesion and correctly localized it in the pelvic abdominal cavity, it is important to follow an algorithmic approach that depends on a combination of clinical, laboratory and imaging features in order to reduce the range of hypotheses and, where possible, arrive at a probable/certain diagnosis. The final goal is to have a tailored therapeutic approach for the patient. Many of the peritoneal lesions, whether primary or secondary, solid or cystic, neoplastic or neoplastic-like, benign or malignant, are diffuse and multifocal. The focal cystic lesions represent a diagnostic challenge, such as mesenteric lymphangioma, mesenteric angiosarcoma, cystic mesothelioma, which constitute the wide range of injuries in this anatomic space. Among solid lesions, it is useful, at the first instance, to detect the presence of particular tissues or structures that can narrow the range of hypotheses, such as macroscopic fat (lipoma, liposarcoma), calcifications (papillary serous carcinoma, carcinoid tumor, sclerosing mesenteritis) or myxoid tissue (that in particular manifest high T2-w signal intensity). Some solid focal lesions instead at MRI demonstrate low T2 signal intensity; among these, we have desmoid (except in cases actively growing or aggressive in which the signal is heterogeneously high on this sequence), which can be poorly marginated or very well circumscribed. These lesions are rare and prevalent in young females. On MRI, a uterine nondegenerated leiomyoma is characteristically homogeneously T2 hypointense [26]. It is reported in the literature that rare retroperitoneal leiomyoma appears as a well-defined homogeneous solid mass with low T2 signal intensity at MRI [27], aspects similar to the same uterine lesions. Also, in our case, the characteristics were similar to those described. Both in the retroperitoneum and in the abdominal cavity, leiomyomas may grow quite large prior to being diagnosed, both for the characteristics of these anatomical spaces and for the nonspecific symptoms that delay the diagnosis [10].

## Conclusions

In the primary pelvic peritoneal masses, the crucial tasks of imaging are the lesion's detection, characterization and staging, if required. As to characterization, T2-w hypointensity of a focal primary pelvic peritoneal mass is an infrequent finding and, especially if associated with a well circumscribed lesion with relative homogeneity without calcifications, which is in keeping with the diagnosis of a fibro-leiomyoma. Therefore MRI proved to be a fundamental tool in our case, allowing us to hypothesize with imaging this neoplasm, which is very rare especially in males, in which it may not be taken into consideration.

## Author Contributions

All authors attest that they meet the currentInternational Committee of Medical Journal Editors (ICMJE) criteria for authorship. D.T., M.A.and V. M. conceptualized the paper; D.T., C.P. L. P. and M.O. evaluated the imaging findings; D.T., L.P., S.A.M. and M.S. drafted the manuscript; and all the authors revised and commented on the paper and approved the final version of the manuscript. All authors have read and agreed to the published version of the manuscript.

## Funding

This research received no external funding.

## Institutional Review Board Statement

The study was conducted according to the guidelines of the Declaration of Helsinki.

## Data Availability Statement

The data presented in this study are available on request from the corresponding author. The data are not publicly available due to privacy restrictions.

## Acknowledgments

No acknowledgments.

## Patient consent

Informed consent was obtained from the subject involved in the study.
